# Psychosis and Catatonia in Fragile X Syndrome

**DOI:** 10.7759/cureus.12843

**Published:** 2021-01-21

**Authors:** Mitra Keshtkarjahromi, Karishma Palvadi, Aayush Shah, Kendall R Dempsey, Silvina Tonarelli

**Affiliations:** 1 Psychiatry, University of Maryland, Baltimore, USA; 2 Psychiatry, Texas Tech University Health Sciences Center El Paso, El Paso, USA

**Keywords:** fragile x syndrome, catatonia, attention deficit hyperactivity disorder (adhd), first episode psychosis, intellectual developmental disorder (idd), antipsychotics, psychiatric comorbidities, child and adolescent psychiatry

## Abstract

Fragile X syndrome is an inherited disorder with an X-linked dominant inheritance pattern that is the most commonly inherited cause of intellectual developmental disorder and has a strong association with autism spectrum disorder. This report describes the case of an 18-year-old male with fragile X syndrome and multiple psychiatric comorbidities who presented with new onset psychosis and catatonia.

## Introduction

Fragile X syndrome (FXS) is the most commonly inherited cause of intellectual developmental disorder (IDD) and it is commonly associated with autism spectrum disorder (ASD) [1-3]. The syndrome has an X-linked dominant inheritance pattern and results from a full mutation (>200 CGC repeats) in the fragile X mental retardation 1 (FMR1) gene located at Xq27.3.2 The FMR1 gene encodes a protein named the fragile X mental retardation protein (FMRP), which is essential for normal brain development. Healthy individuals have 6-54 repeats of the CGG sequence in the 5’ untranslated region of the FMR1 gene [4]. When an individual carries an intermediate number of CGG repeats (55-200) excess FMR1 mRNA is produced and this is considered a pre-mutation allele. The pre-mutation is responsible for fragile X-associated tremor ataxia syndrome (FXTAS), fragile X-associated premature ovarian insufficiency (FXPOI), and fragile X-associated neuropsychiatric disorders [5-9]. In FXS, the number of CGG repeats exceeds 200, and there is a lack of FMRP causing down-regulation of the gamma-amino butyric acid (GABA) A and B receptors and up-regulation of metabotropic glutamate receptors 5 (mGluR5) [10,11]. Males are usually more severely affected by FXS because the affected gene (FMR1) is located on the X chromosome. The typical phenotype of a patient with FXS includes a long face, prominent ears, hyperextensible joints, and post-pubertal macro-orchidism [3]. The syndrome is commonly associated with psychiatric disorders such as attention deficit hyperactive disorder (ADHD), learning disorders, obsessive-compulsive disorder, depression, anxiety, and language function disorders [1,2,9,12-16].

Catatonia is a treatable neuropsychiatric syndrome characterized by abnormal movements, which occurs mainly in the context of an underlying general medical or psychiatric disorder including mood disorders, schizophrenia, and ASD. According to the Diagnostic and Statistical Manual (DSM) for Psychiatric Disorders, 5th edition, the clinical diagnosis of catatonia requires at least three of the following symptoms: stupor (no psychomotor activity), catalepsy (maintaining a passively induced posture), waxy flexibility (slight, even resistance to positioning by the examiner), mutism, negativism (opposition or no response to external stimuli), posturing (spontaneous and active maintenance of a posture against gravity), mannerisms, stereotypies, agitation, grimacing, echolalia, or echopraxia [17]. The underlying neurochemical mechanism of catatonia is similar to the mechanism seen in FXS including GABA hypoactivity and glutamate hyperactivity. However, dopamine dysfunction, a common mechanism of catatonia, is seen in a majority of FXS leading to ADHD during childhood and Parkinsonian symptoms in the elderly [11,15]. The treatment plan usually includes the correction of the underlying disorder; however, benzodiazepines and electroconvulsive therapy (ECT) can also be used as an effective treatment [8,18].

There is a 2.5% risk of developing malignant catatonia and development of neuroleptic malignant syndrome when anti-psychotics are used for the treatment of catatonia [15,18]. Malignant catatonia has a considerable risk of mortality due to cardiovascular instability, nutritional deficiency, functional impairment, and multi-organ failure related complications.

## Case presentation

An 18-year-old Caucasian male with FXS, ADHD, IDD, and ASD diagnosed in childhood was referred by his pediatric neurologist to the psychiatric clinic for evaluation of sudden onset psychosis, catatonia, and behavioral changes. The patient was diagnosed with FXS at birth. Due to having a severe global developmental delay, he was enrolled in an early intervention program and a special education program at his school. The patient has four other siblings, two of his older brothers have FXS and ADHD. The patient’s mother is a carrier for the FMR1 gene and has diagnoses of ADHD, anxiety, and depression. The maternal grandfather was also diagnosed with FXS. The patient’s father has a history of bipolar disorder type 1 and post-traumatic stress disorder. In the month prior to psychiatric evaluation, the patient presented with agitation, aggressiveness, and anger towards family members leading to the patient being isolated in his room. Irritability, insomnia, crying spells, and mood swings were also present. Auditory and visual hallucinations were reported. The patient was seeing people around him and he was talking to them. He also had a fear of someone being behind his back. The patient experienced a loss of basic learned skills including holding a pen, writing, and drawing.

Catatonia including standing in one position with limited movements and limited blinking, staring at the ceiling for prolonged periods of time, waxy flexibility, posturing, stereotypies, echolalia, mutism, and cog-wheel rigidity were noticed. According to his parents, the patient used to be a “sweet, friendly boy” who now is “a very different kid” who is very difficult to manage at home.

Before the psychiatric evaluation, the patient was hospitalized for 12 days in a medical hospital. Vital signs were normal. On neurological examination, severe cog-wheel rigidity in all extremities, bilateral downward-pointing toes, bilateral hands clenched in flexed position, and stiffness with rigidity of the entire body were noted. During the hospital course, all the labs and imaging were normal as can be seen in Table 1. Treatment included a benzodiazepine challenge test, quetiapine, and diphenhydramine with no improvements. The patient was discharged with outpatient psychiatric follow-up.

**Table 1:** Results of Diagnostic Testing

Labs/Imaging	Results
Complete Blood Count	Normal
Complete Metabolic Panel	Normal
Thyroid Stimulating Hormone	Normal
Estimate Sedimentation Rate	Normal
C-Reactive Protein	Normal
Urine Drug Screening	Normal
Blood Toxicology	Normal
Electroencephalography x2	Normal
Brain and Spinal Magnetic Resonance Imaging	Normal
Abdominal Computerized Tomography	Normal
Electrocardiography	Sinus Tachycardia

Course and treatment 

On his first visit to the psychiatric clinic, the patient had been taking quetiapine 150 mg daily and lorazepam 4 mg daily for 30 days without any improvement. On examination, patient was noted to have cog-wheel rigidity, muscle stiffness, and dystonia. He was internally stimulated, and his mood and affect were irritable and anxious. Lack of cooperation and rapport was seen. He was complaining of low back pain due to severe dystonia in his back and chest.

Lorazepam was changed to clonazepam 1 mg twice a day per oral, divalproex sodium 250 mg per day was started, and quetiapine was continued.

On his two-week follow-up, the patient had mild improvement, but he was still isolated from the family due to aggression. Due to the patient's continued symptoms, the patient’s mother decided to initiate lisdexamfetamine 50 mg daily from patient’s older brother’s prescription, which significantly improved the patient’s aggressive behavior. She felt comfortable making this change as she has two other children with the same diagnoses; however, she did report this medication change immediately and has been compliant with her sons' medications after this with no concern for abuse. She was counseled at the follow-up appointment that lisdexamfetamine is a Schedule II substance and should not be started without consultation with a physician. Due to the improvement of symptoms, the dose of divalproex sodium was titrated up to 1000 mg daily and clonazepam was decreased to 0.5 mg twice daily, while quetiapine 150 mg daily and lisdexamfetamine 50 mg daily were continued as before.

At his four-week follow-up, the patient was on the combination of lisdexamfetamine, divalproex sodium, quetiapine, and clonazepam 2 mg daily with marked improvements in psychosis, catatonia, behavioral change, aggression, irritation, and insomnia. His parents reported that the patient was back to his normal self. The patient was no longer isolated from his family and was participating in social activities with them. On physical and mental examination, there were no signs of dystonic positioning, but mild cog-wheel rigidity continued to be noted in the upper extremities. The patient was following commands and had good eye contact with appropriate affect.

The patient continues to be monitored at the psychiatric clinic regularly and has not had a recurrence of symptoms.

## Discussion

FXS has a variety of comorbidities including ADHD, ASD, IDD, and other neuropsychiatric disorders (depression, anxiety, etc.). Psychosis is seen in less than 10% of those with FXS. Catatonia can be present in FXS if it is comorbid with ADHD, ASD, IDD or any other mood disorder, but there is not enough data to support exclusive relationship between the two. The underlying mechanism of catatonia has been identified due to changes in three hormonal activities. These include dopamine hypoactivity, GABA hypoactivity, and glutamate hyperactivity [11,15]. Moreover, the comorbidity between FXS and the development of ADHD in early childhood has been shown to be due to dopamine dysfunction.

Catatonia is increasingly recognized at a rate of 4%-17% in adolescents and adults with ASD [8]. Although the diagnosis of catatonia in ASD is clinical, it may be further supported by a benzodiazepine challenge test, which is known to result in marked and temporary improvement.

The association of catatonia and ADHD is a rare condition and the data published about this association is scarce. The symptoms of excited catatonia can overlap with the symptoms of ADHD, as research has shown improvement in hyperactivity, impulsivity, and inattention with lorazepam [12].

On the other hand, the association between bipolar disorder and catatonia is well known and ECT is an effective treatment used mainly for patients resistant to benzodiazepines [19]. Therefore, it is crucial to consider bipolar disorder in the differential diagnosis in pediatric patients with a diagnosis of ADHD who develop catatonia [20].

Catatonia is a complex condition that is characterized by dysfunction in the regulation of multiple neurotransmitters. There is no standardized pharmacological treatment plan for this heterogenous condition; however, benzodiazepines and ECT remain the most effective treatment at this time. The literature reported a case study of a 25-year-old male with a history of FXS, who developed psychosis and catatonia which were refractory to multiple medications. In that case study, the patient’s catatonia improved with ECT; however he later developed cognitive decline [15]. The current report highlights the association of multiple psychiatric comorbidities in a patient with FXS and the response to a combination of psychotropic medications.

## Conclusions

This is an unusual case report of a patient with FXS and multiple psychiatric comorbidities who presented with new onset catatonia and psychosis. The exact etiology of the catatonia in this case is unclear (secondary to ADHD, IDD, FXS, ASD, or mood disorder); however, the patient had a good response to the combination of divalproex sodium, lisdexamfetamine, quetiapine, and clonazepam. These medications were used to treat the most likely causes of the catatonia.
